# Characterization and Transposon Mutagenesis of the Maize (*Zea mays*) *Pho1* Gene Family

**DOI:** 10.1371/journal.pone.0161882

**Published:** 2016-09-20

**Authors:** M. Nancy Salazar-Vidal, Edith Acosta-Segovia, Nidia Sánchez-León, Kevin R. Ahern, Thomas P. Brutnell, Ruairidh J. H. Sawers

**Affiliations:** 1 Laboratorio Nacional de Genómica para la Biodiversidad (LANGEBIO), Unidad de Genómica Avanzada, Centro de Investigaciones y de Estudios Avanzados del Instituto Politécnico Nacional (CINVESTAV-IPN), Irapuato C.P. 36821, Guanajuato, México; 2 Boyce Thompson Institute for Plant Research, Ithaca, New York 14853-1801, United States of America; 3 Donald Danforth Plant Science Center, St. Louis, Missouri 63132, United States of America; USDA-ARS, UNITED STATES

## Abstract

Phosphorus is an essential nutrient for all plants, but also one of the least mobile, and consequently least available, in the soil. Plants have evolved a series of molecular, metabolic and developmental adaptations to increase the acquisition of phosphorus and to maximize the efficiency of use within the plant. In Arabidopsis (*Arabidopsis thaliana*), the AtPHO1 protein regulates and facilitates the distribution of phosphorus. To investigate the role of PHO1 proteins in maize (*Zea mays*), the B73 reference genome was searched for homologous sequences, and four genes identified that were designated *ZmPho1;1*, *ZmPho1;2a*, *ZmPho1;2b* and *ZmPho1;3*. *ZmPho1;2a* and *ZmPho1;2b* are the most similar to *AtPHO1*, and represent candidate co-orthologs that we hypothesize to have been retained following whole genome duplication. Evidence was obtained for the production of natural anti-sense transcripts associated with both *ZmPho1;2a* and *ZmPho1;2b*, suggesting the possibility of regulatory crosstalk between paralogs. To characterize functional divergence between *ZmPho1;2a* and *ZmPho1;2b*, a program of transposon mutagenesis was initiated using the *Ac*/*Ds* system, and, here, we report the generation of novel alleles of *ZmPho1;2a* and *ZmPho1;2b*.

## Introduction

Phosphorus (P) is an essential nutrient for all plants and a limitation on productivity in many agricultural systems [1]. Current levels of agricultural phosphorus inputs are recognized to be both unsustainable and environmentally undesirable [2]. Rational strategies to improve P efficiency in agricultural systems demand a greater understanding of P relations in crops, both in terms of P uptake from the soil and P translocation and use within the plant.

The protein PHO1 has been characterized in Arabidopsis (*Arabidopsis thaliana*) and rice (*Oryza sativa*) to play a key role both in the export of inorganic P (Pi) to the xylem apoplast for translocation [3] and in the modulation of long-distance signals underlying the P-deficiency response [4]. The Arabidopsis *Atpho1* mutant hypo-accumulates P in the shoots and displays associated symptoms of phosphate deficiency, including reduced growth rate, thinner stalks, smaller leaves, very few secondary inflorescences, delayed flowering and elevated levels of anthocyanin accumulation [3]. In rice, disruption of *OsPHO1;2*, the ortholog of *AtPHO1*, results in a phenotype similar to that of the *Atpho1* mutant [5], suggesting that the two genes are functionally equivalent. Indeed, expression of *OsPHO1;2* in the *Atpho1* background was found to partially complement the mutant phenotype [6]. A feature distinguishing the rice *OsPHO1;2* gene from its Arabidopsis ortholog is the P-regulated production of a *cis*-Natural Antisense Transcript (*cis*-NAT_*OsPHO*1;2_) [5] [7] which acts as a translational enhancer [8].

PHO1 proteins contain two conserved domains: the N-terminal hydrophilic SPX domain (named for the yeast proteins Syg1 and Pho81, and the human Xpr1) and the C-terminal hydrophobic EXS domain (named for the yeast proteins ERD1 and Syg1 and the mammalian Xpr1) [9]. The SPX domain is subdivided into three well-conserved sub-domains, separated from each other by regions of low conservation [7]. SPX domain containing proteins are key players in a number of processes involved in P homeostasis, including fine tuning of Pi transport and signaling by physical interactions with other proteins [7]. E Following the SPX domain there are a series of putative membrane-spanning *α*-helices that extend into the C-terminal EXS domain [9]. In AtPHO1, the EXS domain is crucial for protein localization to the Golgi/*trans*-Golgi network and for Pi export activity, as well as playing a role in the modulation of long-distance root-to-shoot signaling under P limitation [4].

Despite the importance of maize as a staple crop and the dependence of maize production on large-scale input of phosphate fertilizers, the molecular components of maize P uptake and translocation remain poorly characterized [10]. Although it has been possible to identify maize sequences homologous to known P-related genes from other species, functional assignment has been based largely on patterns of transcript accumulation. With the development of accessible public-sector resources, it is now feasible to conduct reverse genetic analyses in maize. Here, we extend the molecular characterization of maize P response by generating mutant alleles of maize *Pho1* genes using endogenous *Activator*/*Dissociation* (*Ac*/*Ds*) transposable elements. The (*Ac*/*Ds*) system consists of autonomous *Ac* elements that encode a transposase (TPase) and non-autonomous *Ds* elements that are typically derived from *Ac* elements by mutations within the TPase gene. Lacking TPase, *Ds* elements are stable, unless mobilized by TPase supplied *in trans* by an *Ac*. *Ac*/*Ds* elements move via a cut-and-paste mechanism [11], with a preference for transposition to linked sites [12] that makes the system ideal for local mutagenesis [13]. To exploit the system for reverse genetics, *Ac* and *Ds* elements have been distributed throughout the genome and placed on the maize physical map, providing potential “launch pads” or mutagenesis of nearby genes [14] [15].

In this study, we identify four maize *Pho1* genes in the maize (var. B73) genome, including two (*ZmPho1;2a* and *ZmPho1;2b*) that we consider co-orthologs of *AtPHO1*. Structure of the *ZmPho1;2a* and *ZmPho1;2b* genes was confirmed experimentally, and accumulation of transcripts characterized in the roots and shoots of seedlings grown in P-replete or P-limiting conditions. Novel insertional alleles of *ZmPho1;2a* and *ZmPho1;2b* are reported, generated using the *Ac*/*Ds* transposon system. Availability of mutant alleles will be central in determining the functional role of *ZmPho1;2a* and *ZmPho1;2b* in maize.

## Materials and Methods

### Identification of maize *Pho1* genes

The AtPHO1 cDNA sequence (GenBank ID: AF474076.1) was used to search the maize working gene set peptide database (www.maizesequence.org) in a BLASTX search performed under the default parameters. Identified maize sequences were in turn used to reciprocally search *Arabidopsis thaliana* (www.phytozome.net). Four sequences were identified encoding proteins with a high level of similarity to AtPHO1: GRMZM5G891944 (chr 3:28,919,073-28,923,871); GRMZM2G466545 (chr 4:171,946,555-171,952,268); a sequence split into the four adjacent gene models GRMZM5G801969,GRMZM5G851655, GRMZM5G815128 and GRMZM5G872499 (chr 5:215,110,603-215,115,635) and GRMZM2G064657 (chr 6:122,577,593-122,582,074). The putative protein sequences were confirmed to contain canonical PHO1 domain structure by PFam analysis (pfam.sanger.ac.uk) and NCBI conserved domains search (www.ncbi.nlm.nih.gov). Additional gene models in the region of *Pho1;2* and their putative orthology relationships were obtained from Ensembl plants (www.plants.ensembl.org).

### Amplification of full length *Pho1;2* cDNAs

Total RNA was extracted using Trizol-chloroform from the roots of 10-day-old B73 seedlings grown under phosphate limiting conditions (sand substrate; fertilized with modified P-free Hoagland solution [16]). 1*μ*g of RNA was used to synthesize cDNA with oligo(dT) primer and *SuperScript III* Reverse Transcriptase (Invitrogen, Carlsbad, CA, USA) in a reaction volume of 20*μ*l. PCR amplification of full length *Pho1;2* cDNAs was performed using using Platinum Taq High Fidelity DNA Polymerase (Invitrogen) under the following cycling conditions: initial incubation at 95°C for 3min, followed by 35 cycles of 94°C for 30sec, 61°C for 30sec and 68°C for 30sec, final extension at 68°C for 5min. Primers used are shown in Table 1. Confirmed gene models were submitted to www.maizegdb.org.

**Table 1:** Primers used in this study for CDS amplification and transcript accumulation.

Target	ID	Primer 5’-3’	Description
*Pho1;2a*	A	MS103- TCGCGGAGGATGGTGAAGTTCT	Full CDS (2463bp)
	H	MS062- GTGTGTCCATTCCTGGAACTCT	
	F	MS002- AGGTGGCCATGAAGTACCTG	Exon 6,7,8,9 and 10 (734bp/1130bp)
	G	MS056- CCTGCATTGCTCTCCAGTAGTAA	
*Pho1;2b*	I	MS013- ATCCCACGATGGTGAAGTTCT	Full CDS (2263bp)
	N	MS077- ACAATTCTCAATCGACCACTAGC	
	L	RS138- AACTCCGTCTCGGTATGGTGGAGTCT	Exon 13, 14 and 3’UTR (383bp/475bp)
	M	RS139- TGACCAGAACGCCTCATGTTATACCC	
*SbPho1;2*	Q	MS105- CATGGACTGGGGCTTCTTAAAC	Exon 13, 14 and 15(293bp/475bp)
	R	MS106- GTAATGGGACAGTCTTCACTGCT	
*cis*-NAT_*Pho*1;2*a*_	B	MS121- GAGCATCCTGATTCCATATCTACC	Flanking putative exon (478bp)
	E	MS120- CCCGTAATGGAAGCTTTTACTG	
*cis*-NAT_*Pho*1;2*b*_	J	MS131- CGTACTGCTGATCGCATGCATA	Flanking putative exon (544bp)
	K	MS132- AGTACGTGATCAGTGATCTACACTC	
*cis*-NAT_*SbPho*1;2_	O	MS162- CTGATCGCTGACAGATGGCCATA	Flanking putative exon (475bp)
	P	MS158- ACCAGCATCCAGCACCCAAAC	
Nested *cis*-NAT	C	MS155- GGAGGTGATGGCGGCGCTGGA	Putative exon (263bp(Pho1;2a/b),
	D	MS163- TCTCGGCGTGCTGGACCTTCTT	266bp(SbPho1;2))
*ZmUBQ*/*SbUBQ*		FWD- CTACAACATTCAGAAGGAGAGCAC	
		REV- TCTGCAAGGGTACGGCCATCC	
*ZmCDK*		FWD- GGAAGGTATGCACAGGACAGAT	
		REV- TTCAGCACAATCTTGGCAAAAC	

### Analysis of *Pho1;2* transcript accumulation

Total RNA was extracted using Trizol-Chloroform from the roots of 10-day-old B73 seedlings grown under P replete (sand substrate; plants fertilized with complete Hoagland solution containing 1mM concentration of PO_4_) or P limiting (sand substrate; fertilized with modified P-free Hoagland solution). cDNA was synthesized as described above. PCR amplification of sense genes was performed using the primers MS002-MS056 (*Pho1;2a*), RS138-RS139 (*Pho1;2b*) and MS105-MS106 (*SbPho1;2*) under the following cycling conditions: initial incubation at 95°C for 5min, followed by 32 cycles of 95°C for 30sec, 63°C for 30sec and 72°C for 1min, final extension 72°C for 5min. PCR amplification of cis-NATs was performed with the primers MS121-MS120 (*cis*-NAT_*Pho*1;2*a*_), MS131-MS132 (*cis*-NAT_*Pho*1;2*b*_) and MS162-MS158 (*cis*-NAT_*SbPho*1;2_) under the following cycling conditions: initial incubation at 95°C for 5min, followed by 38 cycles of 95°C for 30sec, 59°C for 30sec and 72°C for 30sec, final extension 72°C for 5min. Products from the primary PCR reaction were diluted 1:100,000 and used as template for nested PCR with MS155-MS163 primers under the following conditions: initial incubation at 95°C for 5min, followed by 25 cycles of 95°C for 30sec, 58°C for 30sec and 72°C for 15sec, final extension 72°C for 5min. Primers are shown in Table 1. Maize and sorghum poly-ubiquitin (GRMZM2G419891/Sb04g004260) or the maize CDK (GRMZM2G149286) were used as a control, along with amplification from genomic DNA (gDNA) template, using 50ng gDNA in 20*μ*l for 32-cycle reactions and 10ng gDNA in 20*μ*l for 38-cycle reactions. Products were analyzed on 1.5% agarose gels. RNA sequence data for Fig B in S1 File as reported previously [17]

### Transposon mutagenesis

The strategy for *Ac*/*Ds* mutagenesis was as previously described [18] [14]; [15]. Genetic stocks were maintained in the T43 background, a color-converted W22 stock carrying *r1-sc::m3*, a *Ds6-like* insertion in the *r1* locus that controls anthocyanin accumulation in aleurone and scutellar tissues [19]. The frequency of purple spotting in the aluerone resulting from somatic reversion of *r1-sc::m3* was used to monitor *Ac* activity [20]. Donor *Ac* and *Ds* stocks were selected from existing collections [14] [15]: the element *bti31094::Ac* is placed on the B73 physical map 650.8Kb from *ZmPho1;2a*; the element *I.S06.1616::Ds* is inserted in intron 13 of *ZmPho1;2b*, and was subsequently designated *ZmPho1;2b-m1::Ds*. To generate a testcross population for mutagenesis of *ZmPho1;2a*, 207 individuals homozygous for *bti31094::Ac* were crossed as females by T43, and rare, finely spotted progeny kernels were selected for screening. To re-mobilize the *Ds* element *I.S06.1616::Ds* within *ZmPho1;2b*, homozygous *ZmPho1;2b-1::Ds* individuals carrying the unlinked stable transposase source *Ac-Immobilized* (*Ac-im*) [21], were used as males to pollinate T43, and coarsely spotted progeny kernels were selected for screening.

To identify novel *Ac*/*Ds* insertions in *ZmPho1;2a* and *ZmPho1;2b*, selected kernels were germinated in the greenhouse and DNA was isolated from pools of 18 seedlings. The candidate gene space was explored by PCR using a range of gene-specific primers in combination with “outward-facing” *Ac*/*Ds*-end primers. All primers are listed in Table 2. PCR reactions contained 400ng gDNA and.25*μ*M of each primer. Reactions for *ZmPho1;2a* were performed using Platinum Taq High Fidelity DNA Polymerase (Invitrogen) under the following cycling conditions: denaturation at 94°c for 4 min; 30 cycles of 94°c for 30 sec, 58°c for 30 sec, 68°c for 3min 30 sec; final extension at 68°c for 10 min. Reactions for *ZmPho1;2b* were performed using Kapa Taq DNA polymerase (Kapa Biosystems, Wilmington, Massachusetts, USA) under the following cycling conditions: denaturation at 95°c for 5 min; 35 cycles of 95°c for 30 sec, 58°c for 30 sec, 72°c for 3min 30 secs; final extension at 72°c for 5 min. Positive pools were re-analyzed as individuals, following the same cycling conditions. PCR reactions were analyzed on 1.5% agarose gels. Products from positive individuals were purified using the QIAquick PCR Purification Kit (Qiagen, Hilden, Germany), ligated into pGEM T-easy vector (Promega, Fitchburg, Wisconsin, USA) and sequenced. Genotyping of *Zmpho1;2a-m1::Ac* was performed by amplification of fragments spanning the insertion with specific-*Ac* primers (MS124-MS052, MS124-JRS01 and MS052-JGp3; see Table 2), using Kapa Taq DNA polymerase (Kapa Biosystems), under the following cycling conditions: denaturation at 95°c for 5 min; 35 cycles of 95°c for 30 sec, 58°c for 30 sec, 72°c for 3min 30 secs; final extension at 72°c for 5 min. Somatic excision events were identified by *Bse*YI (New England BioLabs, Ipswich, Massachusetts, USA) digest of MS124-MS052 PCR products, according to the manufacturer’s protocol.

**Table 2:** Primers used in this study for genotyping.

Target	ID	Primer 5’-3’	Description
*ZmPho1;2a*		MS069- ACCTTTCTACACTGCCTGTACC	1st fragment (1244bp)
		MS044- GTTCGACCTACCTAACATGGACT	
		MS043- GTTTGTACGTACCCATGCCGTAT	2nd fragment (1577bp)
		MS048- GGAAGGGAAGTACCTTGTCAGAG	
		MS047- CTCCCTCAATGTGAAGGCTTT	3rd fragment (2835bp)
		MS062- GTGTGTCCATTCCTGGAACTCT	
*ZmPho1;2b*		MS085-CTCATTTGTTTCCAGTTTCTCTCC	1st fragment (Promoter—2981bp)
		MS028- AGCTAGCTACCTGACATGGACT	
		MS027- CAGGAGCAAGAGTTTGTGGAGA	2nd fragment (2003bp)
		MS016- GTGCTGGAGAAGTCGAAGATG	
		MS033- GTTCACAGGCACATTTGTGTC	3rd fragment (2088bp)
		MS077- ACAATTCTCAATCGACCACTAGC	
*Zmpho1;2a’-m1.1*	S	MS124- CACACTCATCATCTGAACAAAGCAAG	flanking *Ac* excision (421bp)
	T	MS052- GCATCCTAATAAAGCCTGGAAGA	
*Ac*/*Ds*		JGp3-ACCCGACCGGATCGTATCGG	Transposon primers
		JRS01-GTTCGAAATCGATCGGGATA	

To generate footprint alleles, individuals homozygous for *Zmpho1;2a-m1::Ac* were crossed as males to T43 females. Rare, non-spotted progeny kernels were selected and screened for excision by PCR amplification across the site of *Ac* insertion using primers MS124-MS052 under the following cycling conditions: denaturation at 95°c for 5 min; 35 cycles of 95°c for 30 sec, 58°c for 30 sec, 72°c for 3min 30 secs; final extension at 72°c for 5 min. PCR products from each individual were purified using the QIAquick PCR Purification Kit (Qiagen), ligated into pGEM T-easy vector (Promega) and sequenced. *Ac* excision was confirmed by *Bse*YI digestion as described above.

## Results

### The maize genome contains four *PHO1* homologs

**Fig 1:**
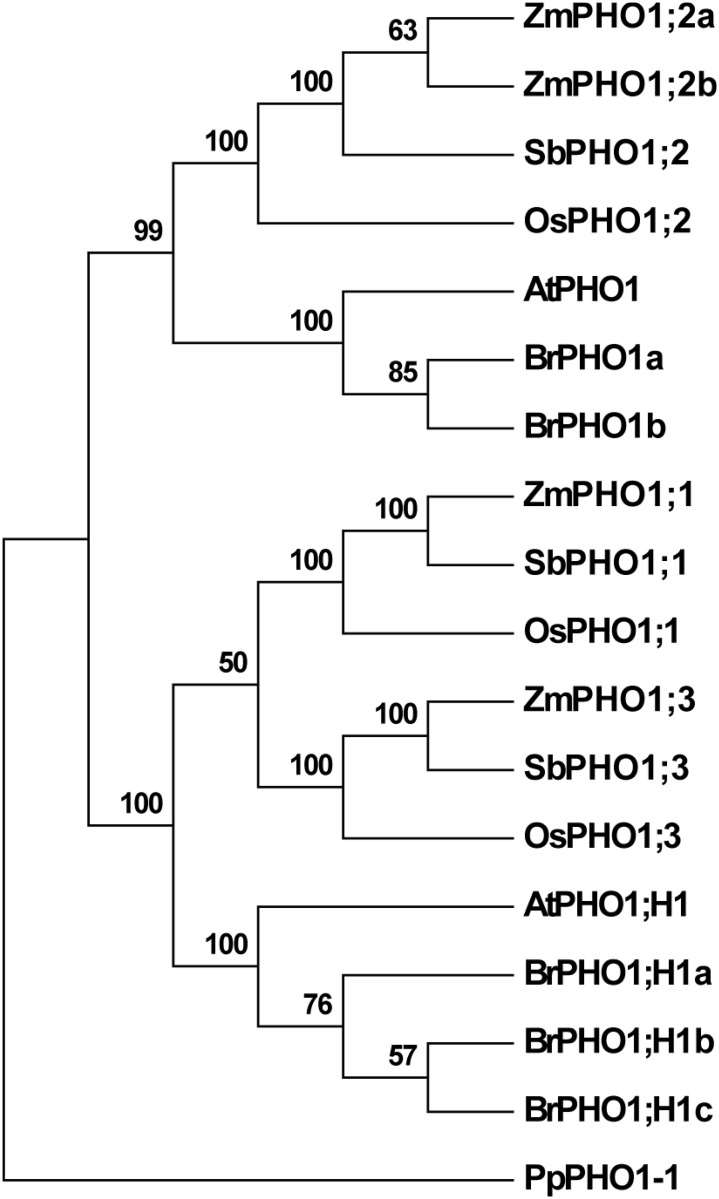
Maximum likelihood reconstruction of the complete set of maize (Zm), sorghum (Sb) and rice (Os) PHO1 proteins along with selected PHO1 proteins of Arabidopsis (At) and canola (Br). The moss (*Physcomitrella patens*) protein PpPHO1-1 is shown as an outgroup. Bootstrap support shown as a percentage at the nodes.

To identify maize *PHO1* genes, the B73 reference genome (B73 RefGen_v3; www.maizegdb.org) was searched to identify gene models whose putative protein products exhibited a high degree of similarity to the *Arabidopsis* protein AtPHO1 (Table 3). Four such maize gene models were identified, and, on the basis of similarity to previously annotated rice genes [5], designated *ZmPho1;1* (GRMZM5G891944), *ZmPho1;2a* (GRMZM2G466545), *ZmPho1;2b* (split across the gene models GRMZM5G801969, GRMZM5G851655, GRMZM5G815128 and GRMZM5G872499) and *ZmPho1;3* (GRMZM2G064657). To investigate orthology among *Arabidopsis thaliana* and grass *PHO1* genes, additional sequences were identified from sorghum (*Sorghum bicolor*) and canola (*Brassica rapa*), and used to generate a multiple alignment and distance tree (Fig 1).

**Table 3:** Maize*Pho1* genes and corresponding*Arabidopsis* and rice orthologs.

*At* Gene	*Os* Gene	*Zm* Gene	*Zm* Gene Model	*Zm* Position
*AtPHO1*	*OsPho1;2*	*ZmPho1;2a*	GRMZM2G466545	chr 4:171,946,555-171,952,268
*AtPHO1*	*OsPho1;2*	*ZmPho1;2b*	GRMZM5G801969	chr 5:215,110,603-215,115,635
			GRMZM5G851655	
			GRMZM5G815128	
			GRMZM5G872499	
*AtPHO1;H1*	*OsPho1;1*	*ZmPho1;1*	GRMZM5G891944	chr 3:28,919,073-28,923,871
*AtPHO1;H1*	*OsPho1;3*	*ZmPho1;3*	GRMZM2G064657	chr 6:122,577,593-122,582,074

The gene *ZmPho1;2b* is split across four adjacent annotated gene models

From *Arabidopsis*, only the proteins AtPHO1 and AtPHO1;H1 were included in the analysis, leaving aside a large clade of divergent functionally distinct PHO1 proteins that are specific to dicotyledonous plants [22]. The analysis supported the previously reported divergence of PHO1 and PHO1;H1 clades, dating from before the divergence of monocotyledonous and dicotyledonous plants [5, 22]. Within the PHO1;H1 clade, a duplication event was observed specific to the grasses in the analysis. As a result, the three grass species each contain two co-orthologs of AtPHO1;H1—encoded by the genes annotated *Pho1;1* and *Pho1;3*. We observed also an expansion of the PHO1;H1 clade in canola, although this expansion is lineage specific, and there is no indication that PHO1;H1 was not encoded by a single gene at the base of this clade. The PHO1 clade itself contains the products of single-copy *PHO1*/*PHO1;2*sequences in all species in our analysis, with the exception of a lineage-specific duplication in maize. As a consequence, the paralogous maize genes *ZmPho1;2a* and *ZmPho1;2b* are considered to be co-orthologos to *AtPHO1*.

### *ZmPho1;2a* and *ZmPho1;2b* show features of syntenic paralogs retained following to genome duplication

**Fig 2:**
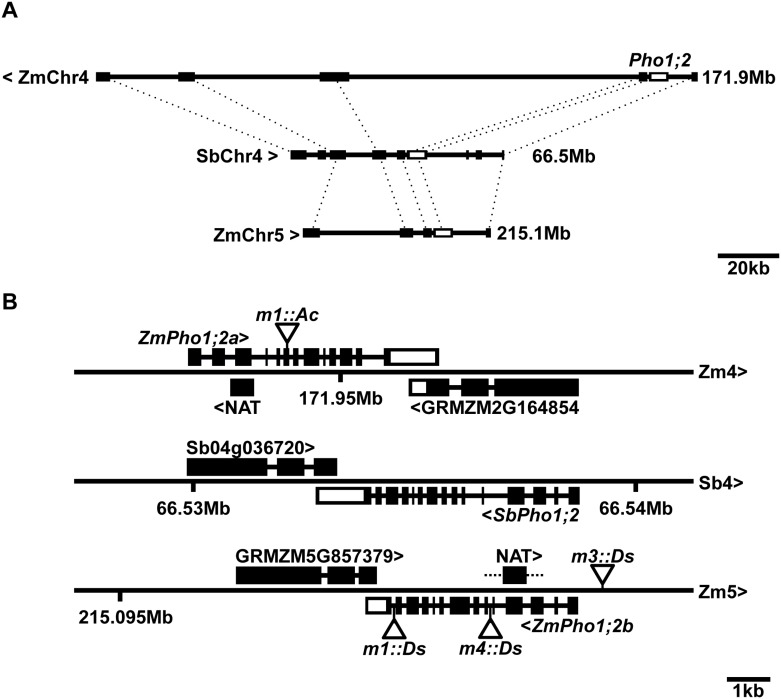
Microsynteny among *Pho1;2* loci A) Annotated genes (filled boxes) in the region of*SbPho1;2* on Sorghum chromosome 4 (SbChr4) and candidate orthologs on maize (B73) chromosomes 4 (ZmChr4) and 5 (ZmChr5). Orthologous genes are connected by dashed lines. *Pho1;2* genes on the three chromosomes shown in unfilled boxes. Regions shown to scale, the right hand position indicated. SbChr4 and ZmChr5 run left to right, ZmChr4 right to left. B) *Pho1;2* gene models on ZmChr4, SbChr4 and ZmChr5. Exons are shown as boxes, coding regions filled, UTR unfilled. Putative anti-sense transcripts associated with *ZmPho1;2a* and *ZmPho1;2b* shown as filled boxes (NAT). Angle brackets indicate the direction of transcription. The maize syntenic paralog pair GRMZM2G164854/GRMZM5G853379 and their sorghum ortholog are also shown. Triangles indicate the position of *Activator* and *Dissociation* insertion. Refer to Table 4 for full description alleles. Shown to scale.

The high degree of sequence similarity (85% protein identity) between *ZmPho1;2a* and *ZmPho1;2b* suggests that they result from a recent gene duplication event. It has been hypothesized that the last whole genome duplication (WGD) event in maize occurred between 5 and 12 million-years-ago, sometime after divergence from the sorghum lineage, as the result of polyploidization [23]. The observation that *Pho1;2* is a single copy sequence in both rice and sorghum is consistent with the maize duplication arising during this most recent WGD. Further inspection revealed that the two genomic regions carrying maize *Pho1;2* genes are both syntenic to the sorghum region carrying *SbPho1;2*, and that the two maize regions have been assigned previously to distinct pre-tetraploid ancestral genomes (Chr4:168,085,162…179,711,318 to sub-genome 2; Chr5:208,925,180…217,680,842 to sub-genome 1; [23]). The return of the maize genome to a diploid organization following WGD has been accompanied by the loss of the majority of duplicate genes through a process known as fractionation [23]. In certain cases, however, pairs of syntenic paralogs have been retained. The genomic region surrounding the *Pho1;2* genes exhibits a number of such candidate pairs in addition to *Pho1;2a* and *Pho1;2b* (Fig 2A), providing ample evidence of micro-synteny between the regions. In both sorghum and maize, *Pho1;2* genes are adjacent to a putative WD40 protein encoding gene, present on the opposite strand and partially overlapping the annotated 3’ UTR region of the *Pho1;2* sequence (Fig 2B), a feature not observed in the other maize or sorghum *Pho1* paralogs.

### Transcripts encoded by *ZmPho1;2a* and *ZmPho1;2b* accumulate preferentially in the roots

**Fig 3:**
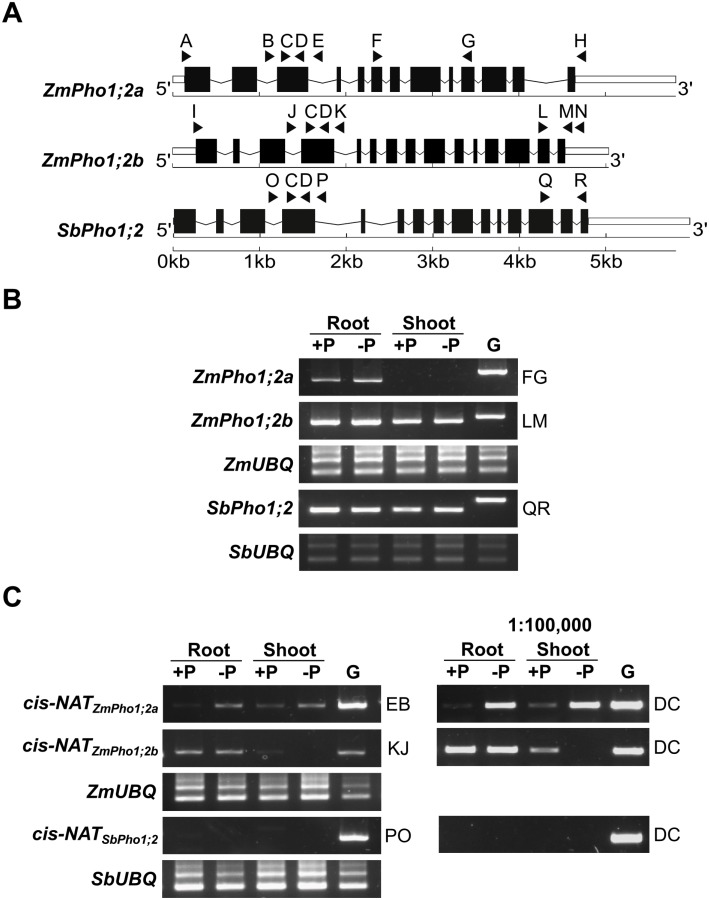
Accumulation of *Pho1;2* sense and antisense transcripts under contrasting phosphate conditions. (A) Schematic of *Pho1;2* genes indicating the position of primers (solid triangles; see Table 1). (B) Amplification of fragments corresponding to the mature sense-RNAs of *ZmPho1;2a*, *ZmPho1;2b* and *SbPho1;2* from oligo-dT primed cDNA prepared from roots and shoot of 10-day-old seedlings, fertilized with Hoagland solution adjusted to 1mM (+P) or 0mM (-P) inorganic phosphate. Primer pairs used for amplification indicated by letter codes on the right-hand side of panel. (C) Amplification of fragments corresponding to putative anti-sense transcripts encoded by *ZmPho1;2a*, *ZmPho1;2b* from cDNA as Panel A. Primary PCR (left) and nested PCR performed from 1:100,000 dilution of primary reaction (right). Amplification of *ZmUBQ* and *SbUBQ* fragments and amplification from genomic DNA template (G) were used as controls. Primer pairs indicated as in (B).

To determine the pattern of accumulation of transcripts encoded by *ZmPho1;2a* and *ZmPho1;2b*, RT-PCR was used to amplify gene-specific fragments of the two genes from cDNA generated from roots or leaves of 10-day-old seedlings (B73) grown in sand, watered with either complete (+P) or a modified P-free Hoagland solution (-P) (Fig 3A and 3B). The pattern of transcript accumulation was similar for *ZmPho1;2a* and *ZmPho1;2b*: stronger amplification in roots than shoots; no difference between P replete and P limiting conditions. The strength of amplification, however, was greater for *ZmPho1;2b* than *ZmPho1;2a*, the latter being detectable in shoots only with a high number of PCR cycles (Fig A in S1 File). The accumulation of *SbPho1;2* transcripts in sorghum (BTx623) seedlings was also examined, under the same growth conditions. Transcripts of *SbPho1;2* accumulated in a pattern similar to that observed for the maize genes, suggesting this pattern to be the ancestral, pre-WGD, state. To investigate further the possibility of divergence at the level of transcript abundance between *ZmPho1;2a* and *ZmPho1;2b*, we examined a previously published transcriptome data set [17]. Transcriptome data confirmed both the pattern of transcript accumulation with respect to tissue type and P availability and the difference in the level of transcript accumulation between the two maize *ZmPho1;2* paralogs (Fig B in S1 File). In addition, transcriptome data indicated *ZmPho1;1* transcripts to accumulate constitutively to low levels and *ZmPho1;3* transcripts to accumulate preferentially in leaves rather than roots. Further gene-specific PCR primers were designed and used to amplify the complete *ZmPho1;2a* and *ZmPho1;2b* cDNAs that were sequenced to confirm the gene-model structure.

### *ZmPho1;2a* and *ZmPho1;2b* are associated with phosphate-regulated putative *cis*-Natural Anti-sense Transcripts

Although we did not observe differential accumulation of *ZmPho1;2* transcripts with respect to P availability, it has been reported that the *Pho1;2* in rice is largely regulated at the post-transcriptional level by a P-regulated *cis*-Natural Anti-sense Transcript *cis*-NAT_*OsPho*1;2_ [5]; [8]. The *cis*-NAT_*OsPho*1;2_ transcript has been shown to act as a translational enhancer, and has been proposed to act by direct interaction with the sense transcript [8]. The rice *cis*-NAT_*OsPho*1;2_ initiates in Intron 4 of *OsPho1;2* and extends into the 5’ UTR region [8]. A putative *ZmPho1;2a* anti-sense sequence is annotated in the maize reference genome in a homologous position to the rice transcript (www.maizegdb.org), although, on the basis of cDNA evidence, the transcript is considerably shorter than *cis*-NAT_*OsPho*1;2_, being trunctated at the 3’ end and extending only as far as Intron 2 of *ZmPho1;2a* (Fig 2B). No paralogous sequence has been annotated associated with *ZmPho1;2b*.

To investigate the presence of *cis*-NAT transcripts associated with *ZmPho1;2a* and explore the possibility that a paralogous *cis*-NAT transcript might be generated from *ZmPho1;2b*, gene-specific primers were designed to the introns flanking the homologous Exons 4 and 3 of *ZmPho1;2a* and *ZmPho1;2b*, respectively. These primers were used to attempt to amplify products from cDNA prepared from seedling root and leaves as described above. Products of the predicted size were successfully amplified using both *ZmPho1;2a* and *ZmPho1;2b* primer sets, consistent with the accumulation of *cis*-NATs (Fig 3B). No products were amplified from no-RT control samples (data not shown). Putative *cis*-NAT products were sequenced and confirmed to originate from the *ZmPho1;2a* and *ZmPho1;2b* genes. There was no evidence of the accumulation of alternatively or partially spliced transcripts during the previous amplification of full length *Pho1;2* cDNAs.

The accumulation of the putative *cis*-NAT_*ZmPho*1;2*a*_ was observed to be induced under -P conditions in both roots and leaves. In contrast, the putative *cis*-NAT_*ZmPho*1;2*b*_ transcript showed no response to P availability in roots and reduced accumulation in shoots under -P (Fig 3B), providing evidence of functional divergence between paralogs at the level of anti-sense production. Interestingly, using the approach we employed in maize, we found no evidence of an equivalent *cis*-NAT associated with *SbPho1;2* (Fig 3B), although additional experiments will be required to rule out the possibility that anti-sense transcripts might initiate from other regions of the sorghum gene.

### Transposon mutagenesis of *ZmPho1;2a* and *ZmPho1;2b*

To investigate functional divergence between *ZmPho1;2a* and *ZmPho1;2b*, we initiated a program to mutagenize both loci using the endogenous *Activator*/*Dissociation* (*Ac*/*Ds*) transposon system. *Ac* and *Ds* elements show a strong preference for linked transposition, allowing a given element to be used for mutagenesis of nearby candidate genes. Once established, it becomes possible to generate multiple alleles from a single test-cross population.

**Fig 4:**
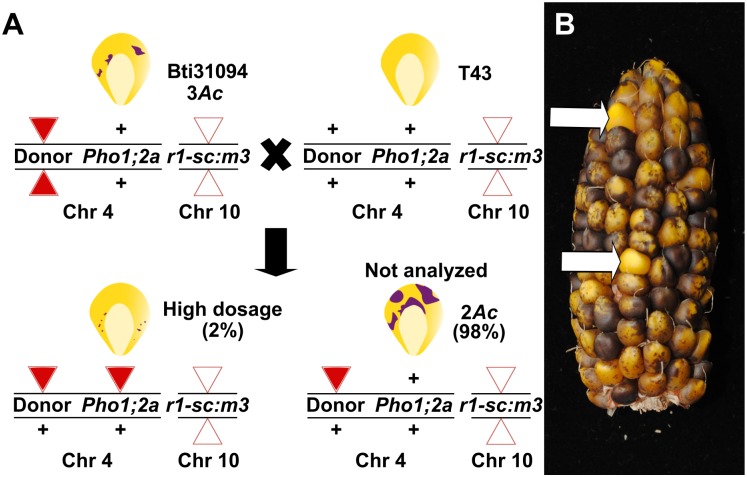
*Ac* mutagenesis of *ZmPho1;2a*. (A) Genetic strategy to mobilize the donor element *bti31094::Ac*. Individuals homozygous for the donor element, displaying the characteristic 3 *Ac* dosage pattern of excision from *r1-sc:m3* in the triploid aleurone, were crossed as females by T43. A small proportion (~2%) of the progeny kernels were finely spotted, indicating high *Ac* dose as a result of replicative transposition, and were screened for potential re-insertion into the target gene. (B) Two high *Ac* dose kernels (white arrows) highlighted among the largely 2 *Ac* dose progeny of a typical test cross ear.

**Fig 5:**
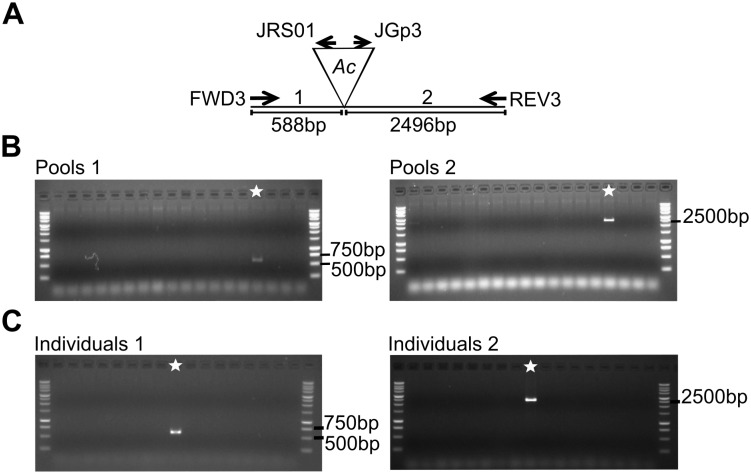
PCR screening for novel *Ac* insertion in *ZmPho1;2a*. (A) Schematic of Zmpho1;2a-m1 allele. Reaction 1 (588bp expected product) and reaction 2 (2496bp) are indicated. (B) PCR reactions using gene and *Ac* specific primers to amplify potential products from DNA pools. Pool with a star shows amplification in complementary reactions 1 and 2. (C) PCR reactions using individual DNA. Amplification from a positive individual in complementary reactions 1 and 2 indicated with a star.

**Fig 6:**
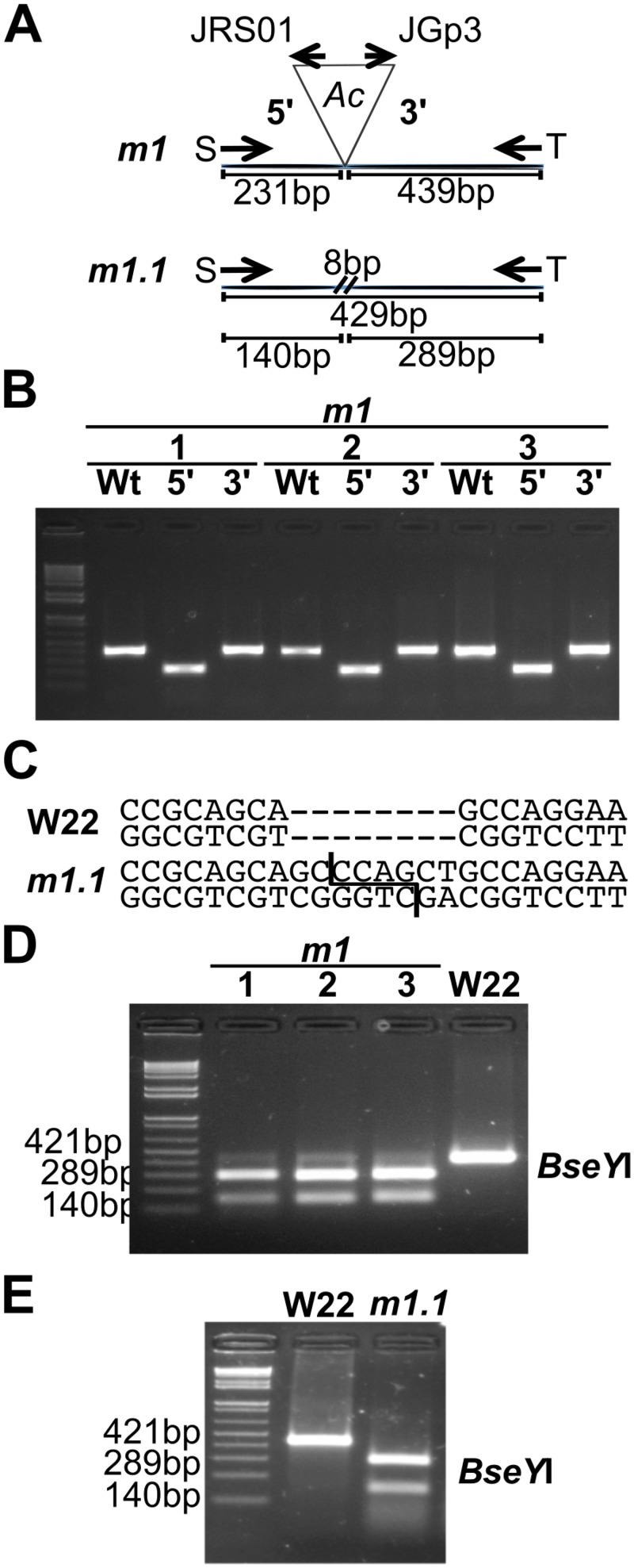
Recovery of *Ac* excision events from *Zmpho1;2a-m1*. (A) Schematic of primer position and fragment lengths corresponding to *Zmpho1;2a-m1* (*m1*) and derived excision events (*m1.1*). (B) Amplification of apparent wild-type (Wt) and flanking products (5’, 3’) from three different individuals genotypically homozygous *Zmpho1;2a-m1* on the basis of kernel phenotype and pedigree. (C) Generation of a *Bse*YI cutting site, not present in wild-type (W22), as the result of an insertion of 8bp following excision of *Ac* from *Zmpho1;2a-m1*. (D) *Bse*YI digestion of products amplified in (B) from three genotypically *Zmpho1;2a-m1* homozygous individuals and a wild-type (W22) individual. (E) *Bse*YI digestion of products amplified with the insertion spanning primers MS124/MS052 from a wild-type individual (W22) and an individual homozygous for the stable germinal excision allele *pho1;2a’m1.1*. 1Kb plus DNA ladder (Invitrogen) is loaded on the first lane in B,C and E.

To mutagenize *ZmPho1;2a*, we recovered 1082 novel transposition events from an *Ac*element (*Bti31094::Ac*) located 650.8kb upstream, selecting rare high *Ac* dosage kernels from a testcross population (Fig 4). A PCR-based strategy was designed to screen for reinsertion of *Ac* into *ZmPho1;2a*: the gene was divided into three overlapping fragments, and, allowing for both possible orientations of *Ac* insertion, we performed a total of 12 reactions to cover the gene space, screening first pools of 18 seedlings, and subsequently the individuals constituting positive pools. Putative insertions were re-amplified using DNA extracted from a second seedling leaf to reduce the probability of selecting somatic events. Using this strategy, we recovered a novel germinal *Ac* insertion in Exon 6 of *ZmPho1;2a* (*Zmpho1;2a-m1::Ac*) (Fig 5). Left and right flanking border fragments were amplified and sequenced, confirming the exact location of the element and identifying an 8bp target site duplication (TSD; AGCCCAGG) typical of *Ac* insertion. Analysis of progeny derived from the original positive plant revealed a high-frequency of somatic excision from *Zmpho1;2a-m1::Ac*: apparently wild-type fragments were routinely amplified spanning the insertion site from individuals selected on the basis of kernel spotting pattern to be genotypically homozygous for *Zmpho1;2a-m1::Ac*. Sequencing of these products revealed them to be the result of somatic *Ac* excision (data not shown), as indicated by a short, typically 8bp, insertion adjacent to the excision site. Excision of *Ac* from *Zmpho1;2a-m1::Ac* coupled with a TSD is predicted to generate a *Bse*YI restriction site (CCCAGC), providing a means to confirm events by digestion of PCR products (Fig 6A, 6B and 6D).

**Fig 7:**
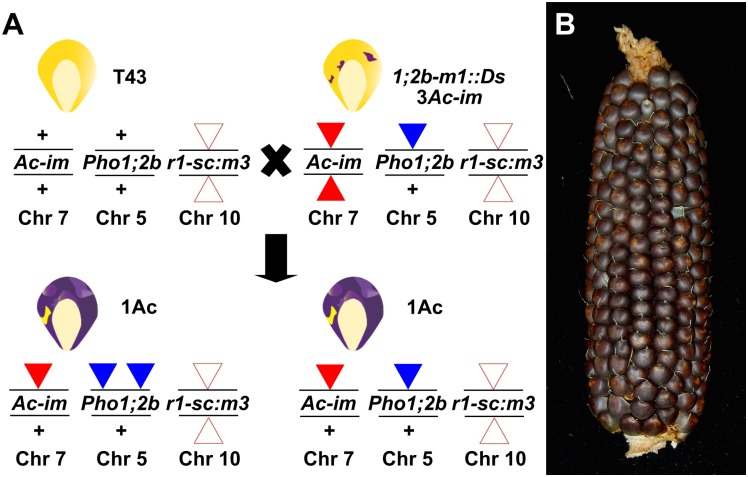
*Ds* mutagenesis of *ZmPho1;2b*. (A) Genetic strategy to re-mobilize the *Ds* element *ZmPho1;2b-m1::Ds*. Individuals carrying *ZmPho1;2b-m1::Ds* and the stable transposase source *Ac-im* were crossed as males to T43. All progeny were screened as rare kernels carrying transposed *Ds* were indistinguishable from other progeny on the basis of aluerone phenotype. (B) Ear carrying kernels with one copy of *Ac-im* in the chr7 expressed in the triploid aleurone.

To identify novel *Ds* insertions in *ZmPho1;2b*, *I.S06.1616::Ds* (designated *Zmpho1;2b-m1::Ds*), inserted in intron 13 of the target gene, was re-mobilized. Plants homozygous for the *Zmpho1;2b-m1::Ds* allele did not present any observable phenotype and RT-PCR analysis of transcript accumulation indicated such plants to accumulate correctly-spliced transcript to normal levels (data not shown). To derive further alleles, individuals homozygous for *Zmpho1;2b-m1::Ds* carrying the unlinked stable transposase source *Ac-immobilized* (*Ac-im*; [21]) were crossed as males to T43 (Fig 7), and test-cross progeny screened using a strategy comparable to that employed in the mutagensis of *ZmPho1;2a*. Two novel *Ds* insertions were identified, one in the promoter region, 591bp upstream of the ATG (*Zmpho1;2b-m3::Ds*) and the second in intron 5 (*Zmpho1;2b-m4::Ds*). Sequencing of the region flanking each novel insertion identified the expected 8bp TSD (Table 4).

**Table 4:** Transposon insertion alleles of maize *Pho1;2* genes.

Alleles	Position (bp)	Description
*pho1;2a-m1::Ac*	2261	*Ac* in Exon 6; TSD: AGCCCAGG
*pho1;2a’-m1.1*	2260	Footprint (CTGCCCAG)from *pho1;2a-m1::Ac* in Exon 6
*pho1;2a’-m1.2*	2260	Footprint (CCCAG) from *pho1;2a-m1::Ac* in Exon 6
*pho1;2b-m1::Ds*	4104	*Ds* in intron 13; TSD: GGTGGGAG
*pho1;2b-m3::Ds*	-591	*Ds* in promoter; TSD: ATCACTAT
*pho1;2b-m4::Ds*	1943	*Ds* Intron 5; TSD: CACACGCT

Position relative to ATG. B73 genomic sequence (RefGen v3) as reference. TSD—target site duplication.

### Derivation of stable derivatives of *ZmPho1;2a*

**Fig 8:**
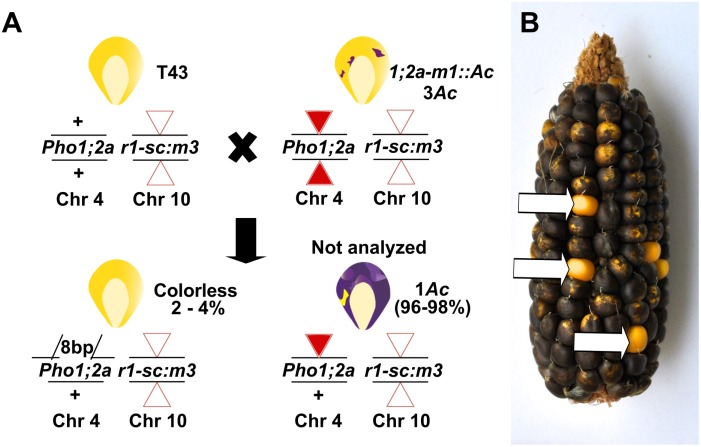
Generation of stable footprint alleles by *Ac* excision. (A) Genetic strategy to re-mobilize to recover stable germinal excision of *Ac* from *ZmPho1;2a-m1::Ac*. An individual homozygous for *ZmPho1;2a-m1::Ac* was crossed as male to T43. Rare colorless kernels were selected for PCR screening. (B) Colorless kernels (white arrows) highlighted among the largely 1 *Ac* dose progeny of a typical test cross ear.

To generate stable “footprint” alleles by *Ac* excision from *Zmpho1;2a-m1::Ac*, a homozygous *Zmpho1;2a-m1::Ac* individual was crossed as a male to multiple T43 females, and resulting colorless progeny screened by PCR amplification across the *Zmpho1;2a-m1::Ac* insertion site (Fig 8). Products of a size consistent with *Ac* excision were cloned and sequenced. Two footprint alleles were identified, one with an 8bp insertion (CTGCCCAG) (*Zmpho1;2a’-m1.1*) and the second with a 5bp insertion (CCCAG) (*Zmpho1;2a’-m1.2*). The region spanning the excision site was re-amplified and digested with the enzyme *Bse*YI, confirming the presence of the footprint (Fig 6A, 6C and 6E). As a result of non-triplet insertion, both *ZmPho1;2a’-m1.1* and *ZmPho1;2a’-m1.2* alleles disrupt the DNA reading frame and are predicted to result in a premature termination of translation (Table 4).

## Discussion

Maize is the most widely grown cereal in the world (ref http://faostat3.fao.org). Much of this cultivated area is P limited. And yet, the molecular basis of P uptake and translocation in maize remains poorly characterized (reviewed in [10]). In this study, we have described the maize *Pho1* gene family and generated novel mutant insertion alleles of the two *ZmPho1;2* genes using the endogenous maize *Ac*/*Ds* transposon system. The genetic material described here initiates the functional analysis of P homeostasis in maize.

The maize *PHO1* family consists of four genes, corresponding to the three gene (*PHO1;1*, *PHO1;2*, *PHO1;3*) structure reported previously in rice (*Oryza sativa*) [5], with the elaboration of a duplication of the maize *ZmPho1;2*. The sorghum *PHO1* family was found also to consist of three genes. The restricted *PHO1* family present in these cereals is in contrast to larger 11-member family of *Arabidopsis* [9]. Specifically, the cereals lack a large clade of *PHO1* related sequences present in *Arabidopsis* that has been implicated in a range of biological functions extending beyond P homeostasis [9, 22, 24]. Indeed, in experiments to complement the *Atpho1* phenotype by expression of other *Arabidopsis* *PHO1* family members, it was only *AtPHO1;H1* that could rescue the mutant [22]. Although phylogenetic analysis and experimental data from *Arabidopsis* and rice suggest all four maize PHO1 to be directly involved in P homeostasis, further work in heterologous systems, and ultimately the analysis of the mutants described here, will be required to determine functional equivalence across species and the biological role in maize.

The lineage leading to maize experienced a tetraploidy event resulting in whole genome duplication (WGD) sometime after the split with the sorghum lineage, 5-12 million years ago [23]. Taking contemporary sorghum to represent the pre-duplication state, immediately following the tetraploid event maize would have carried six *Pho1* genes, represented by three pairs of syntenic paralogs (homeologs). Subsequently, the maize genome has returned to a diploid state through a process of reorganization that has been coupled with extensive fractionation—the loss of one of a pair of syntenic paralogs [25]. Large scale gene loss following WGD appears to be a general trend observed across taxa and across timescales [26]. Gene loss is presumed to be buffered by the presence of functionally equivalent paralogs. Where both paralogs of a syntenic pair are retained, it may indicate either selection or simply incomplete fractionation. The former case would imply functional divergence or a selective advantage of increased dosage. In maize, it is estimated that 3228 pairs of syntenic paralogs have been retained, representing ^~^20% of the total complement of ^~^32,000 total genes, or closer to ^~^10% of the pre-duplication gene set [23] [27]. While gene loss is the more likely outcome following genome duplication, it is difficult to determine the balance of selective gene-by-gene reduction and the largely random loss of larger sections of DNA. Similarly, where a pair of syntenic paralogs are retained, as is the case with *Pho1;2*, it may indicate selection directly on the gene pair or a genomic context that insulates the gene pair from larger scale DNA loss events. It is noticeable that a number of syntenic paralog pairs have been retained close to the *Pho1;2* locus, potentially “hitchhiking” on direct selection to maintain one or more of the adjacent pairs. In the case of the pair GRMZM2G164854/GRMZM5G853379, the two paralogs overlap directly with *Pho1;2* sequence on the opposite DNA strand. Consequently, selection to maintain either the *Pho1;2* or GRMZM2G164854/GRMZM5G853379 pair would protect the adjacent genes from silencing or deletion.

Transcripts of *ZmPho1;2a* and *ZmPho1;2b* were found to accumulate similarly with respect to tissue-specificity and P availability. The absolute level of transcript accumulation, however, was observed to differ between paralogs, consistent with a model of ongoing fractionation in which one member of a paralog pair becomes functionally less significant prior to loss. We did, however, observe regulatory divergence at the level of putative *cis*-NAT transcripts: accumulation of *cis*-NAT_*ZmPho*1;2*a*_ was induced by P limitation, in a manner similar to that observed for*cis*-NAT_*OsPho*1;2_, while *cis*-NAT_*ZmPho*1;2*b*_ accumulation mirrored that of the *ZmPho1;2b* sense transcript, suggesting that, with respect to anti-sense transcript production, it is *ZmPho1;2a* that is maintaining the ancestral, and functionally significant, role. Although, as their name implies, characterized *cis*-NATs act on adjacent protein coding genes, a translational enhancer function, such as that observed in rice for the *PHO1;2* NAT, may allow for *trans* action when, as in maize, two highly similar *ZmPho1;2* paralogs are present. Indeed, one intriguing hypothesis, suggested by our transcript accumulation data, is that in maize there has been sub-functionalization of *PHO1;2*, with the primary production of sense transcripts from *ZmPho1;2b* and the primary production of anti-sense transcripts from *ZmPho1;2a*.

Characterization of the insertional alleles described here will be central in determining the function of *ZmPho1;2a* and *ZmPho1;2b*. We are continuing to mobilize *Ac* and *Ds* elements at the maize *Pho1;2* loci, taking full advantage of the capacity of the system to generate allelic series, impacting variously sense and anti-sense transcripts. Such material will be invaluable in the fine-scale evaluation of regulatory crosstalk and functional redundancy between between *ZmPho1;2* paralogs and, ultimately, the biological role of PHO1 proteins in maize.

## Supporting Information

S1 File (A) Accumulation of *Pho1;2* sense transcripts under contrasting phosphate conditions. Amplification of fragments corresponding to the mature sense-RNAs of *ZmPho1;2a* and *ZmPho1;2b* from oligo-dT primed cDNA prepared from roots and shoot of 10-day-old B73 seedlings, fertilized with Hoagland solution adjusted to 1mM (+P) or 0mM (-P) inorganic phosphate. PCR was run for 27 (27X), 32 (32X) or 37 (37X) cycles. Amplification of *ZmCDK* and amplification from genomic DNA template (G) were used as controls. (B) Accumulation of Pho1;2 sense transcripts detected by RNA sequencing. RNA was extracted from roots and leaves of 21-day old B73 seedlings fertilized with 1000*μ*M (P+) or 10*μ*M (P-) phosphate, and used to generate libraries for RNA sequencing. Data presented as Log2 counts per million (CPM).
(PDF)
