# Supplementary material for: Characterization and Transposon Mutagenesis of the Maize (Zea mays) Pho1 Gene Family
Source: PLoS One. 2016 Sep 20;11(9):e0161882. doi: 10.1371/journal.pone.0161882 (PMC5029887; doi:10.1371/journal.pone.0161882)
Supplement: S1 File — (A) Accumulation of Pho1;2 sense transcripts under contrasting phosphate conditions. Amplification of fragments corresponding to the mature sense-RNAs of ZmPho1;2a and ZmPho1;2b from oligo-dT primed cDNA prepared from roots and shoot of 10-day-old B73 seedlings, fertilized with Hoagland solution adjusted to 1mM (+P) or 0mM (-P) inorganic phosphate. PCR was run for 27 (27X), 32 (32X) or 37 (37X) cycles. Amplification of ZmCDK and amplification from genomic DNA template (G) were used as controls. (B) Accumulation of Pho1;2 sense transcripts detected by RNA sequencing. RNA was extracted from roots and leaves of 21-day old B73 seedlings fertilized with 1000μM (P+) or 10μM (P-) phosphate, and used to generate libraries for RNA sequencing. Data presented as Log2 counts per million (CPM). (PDF) [file pone.0161882.s001.pdf]

Salazar-Vidal et al. Supplementary Figure 1A

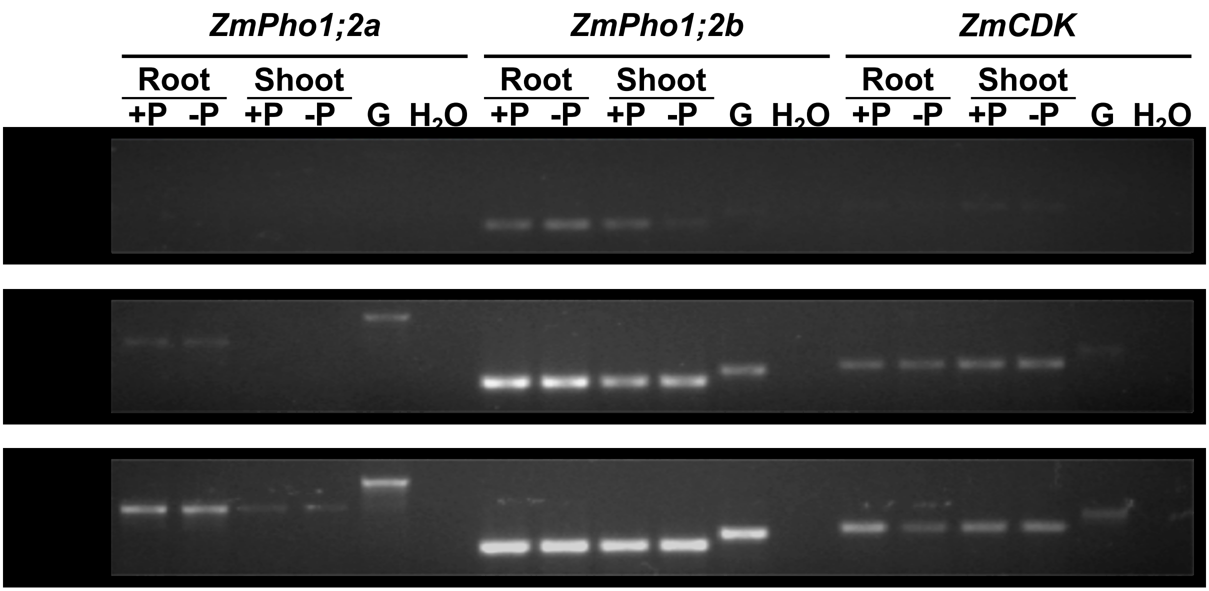

Accumulation of *Pho1;2* sense transcripts under contrasting phosphate conditions. Amplification of fragments corresponding to the mature sense-RNAs of *ZmPho1;2a* and *ZmPho1;2b* from oligo-dT primed cDNA prepared from roots and shoot of 10-day-old B73 seedlings, fertilized with Hoagland solution adjusted to 1mM (+P) or 0mM (-P) inorganic phosphate. PCR was run for 27 (27X), 32 (32X) or 37 (37X) cycles. Amplification of *ZmCDK* and amplification from genomic DNA template (G) were used as controls.

Salazar-Vidal et al. Supplementary Figure 1B

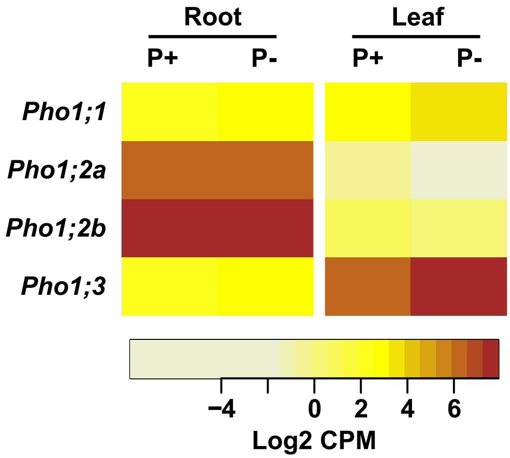

Accumulation of *Pho1;2* sense transcripts detected by RNA sequencing. RNA was extracted from roots and leaves of 21-day old B73 seedlings fertilized with 1000µM (P+) or 10µM (P-) phosphate, and used to generate libraries for RNA sequencing as previously described (González-Muñoz et al., 2015). Data presented as Log2 counts per million (CPM).
